# Perception, knowledge, and consumption pattern of dietary supplement used during COVID-19 pandemic among black Africans: Perspective of Nigerians

**DOI:** 10.1016/j.dialog.2023.100106

**Published:** 2023-02-01

**Authors:** Susan J.A. Holdbrooke, Bamgboye M. Afolabi, Nkiru A. David, Kafilat O. Kareem, Abideen Salako, Oluwagbemiga O. Aina

**Affiliations:** a Nigerian Institute of Medical Research, 6 Edmond Crescent, Yaba, Lagos, Nigeria; b Health, Environment and Development Foundation, Yaba, Lagos, Nigeria

**Keywords:** Black Africans, Consumption, Covid-19, Dietary, Herbal, Micro-nutrients, Supplements, Vitamins

## Abstract

The awareness of the health implication of Covid-19 pandemic marked an increase consumption of various dietary and herbal supplements (DHS) for the deterrence and/or prophylaxis against the novel emerging and infectious disease. However, there is little indication of the usefulness or otherwise of their use in alleviating symptoms of COVID-19.

**Objectives:**

To investigate the pattern and determinants of DHS use among the Nigerian population for the prevention and treatment of COVID-19.

**Design:**

Cross-sectional questionnaire survey. Setting: Older adolescents and adults residing in Nigeria.

**Participants:**

Participants (*n* = 645) residing in the Nigeria were recruited from different geo-political zones and various ethnic groups.

**Primary and secondary outcomes:**

Prevalence and determinants for the use of different DHS for the prevention and treatment of COVID-19 in Nigeria, and sources of information for DHS use.

**Results:**

Most participants (425, 65.9%) believed that dietary supplements are necessary during infectious disease outbreak, but a fewer proportion believed that supplements can be used in conjunction with other drugs to treat Covid-19. Vitamin C was the most known (70.0%) and Vitamin A. The least known (0.3%) dietary supplement Approximately half (50.2%) of the study subjects, more than a third (37.8%) and less than a quarter (22.7%) were aware that Folic acid, vitamin D and vitamin E are DS. Herbal dietary supplements mentioned as known by the study participants included Garlic (46.5%), Ginger (44.7%), Tumeric (36.3%), Moringa (40.0%) and Ginseng (26.3%). Citrus fruit as a DS was recognized by fewer (6.5%) study participants and only 1.6% referred to herbal tea as DHS. In all, 571 (88.5%) of the study participants took DHS during the Covid-19 pandemic with males 1.5 times more likely to take DHS than females (χ^2^ = 3.09, *P*-value = 0.08, OR = 1.54, 95% CI = 0.95, 2.47) during the pandemic. Participants reported lesser consumption of Selenium (27, 4.2%), Iron (20,3.1%), Zinc (61, 9.5%) and calcium (101, 15.7%) to prevent/treat Covid-19. Majority (271, 42.0%) of the study participants mentioned “health worker” as source of information on DHS while 13% mentioned “Social media”. The sociodemographic determinants of DHS practices used to prevent/treat COVID-19 during the pandemic included older age group of 61–70 years, widows, secondary level of education and not employed.

**Conclusions:**

The findings showed widespread use of DHS for the prevention and treatment of COVID-19. The use of DHS in this study was mainly guided by health workers with a marginal role of social media and Mass media. These findings call for a more robust consolidative tactic towards DHS to ensure its proper and safe use.

## 1 Introduction

The outbreak of an unknown origin of pneumonia from Wuhan, Hubei province, China occurred in late 2019. The novel coronavirus, COVID-19 was then discovered to be the aetiology [1,2]. Further studies showed a few neurological symptoms in some patients infected with the severe acute respiratory syndrome coronavirus 2 (SARS-CoV-2) such as headache, languidness, unstable walking, and malaise [3], cerebral hemorrhage [4], and cerebral infarction [5]. The virus has an RNA component covered by viral capsid protein and has a novel genomic mutation, this made it to be resistant to standard medications [6]. Highly contagious with droplets and contact transmission, the virus might stay silent for some few days [7] and then manifests with flu-like symptoms, non-productive coughs, fever, diarrhea, muscle pain, or loss of sense of smell in some cases, severe infection had acute cerebrovascular diseases, impaired consciousness, and skeletal muscle injury [4]. Viruses cause serious outbreaks in all continents leading to difficult morbidities and mortality, and enormous economic burden for the world. In addition, the constant emergence of new serotypes in virus groups that have a high mutation rate and low fidelity for viral replication adds challenges in combatting against these viruses. Viruses are sub-grouped into those containing a lipid envelope and those whose genome is only covered by a protein shell. Enveloped viruses are less stable and more prone to degradation when treated with lipid solvents. Their infection mechanisms are usually based on the presence of fusogenic peptides in the lipid envelope leading to a merge of viral and cellular membranes. Those whose genomes are covered by a protein shell are much more stable and may stay active in wastewaters and on surfaces from several weeks to months. The non-enveloped viruses such as Noro viruses and enteroviruses are therefore causing outbreaks that are difficult to handle. In addition, they show little sensitivity to chemical disinfectants [8,9]. Although Covid-19 broke out from Wuhan, Hubei Province, China on December 8, 2019, it was first reported to the World Health Organization (WHO) on December 31, 2019, and on January 30, 2020, WHO declared the outbreak as a Public Health Emergency of International Concern (PHEIC) [[10], [11], [12]]. About two months later, on March 11, 2020, WHO declared Covid-19 a global pandemic, 11 years after the body declared another infectious disease, H1N1 influenza, a global pandemic in 2009 [13]. The designation “Covid-19” is an acronym of “2019 novel corona virus”, initially called Wuhan virus, the disease that produces severe acute respiratory syndrome coronavirus 2 (SARS-CoV-2). By early October 2021, Covid-19 had already infected over 230 million people globally [14] which shocked the world and caused governments to take decisive measures to curb its spread, prevent infection and reinfection and treat those who are infected. The countries that bore the highest health burden of Covid-19 were United States of America with over 2 million cases, Brazil with over 700,000 cases, India also with approximately 600,000 cases Russia (over 500,000 cases), and in Africa, South Africa (over 54,000 cases) and Egypt (over 38,000 cases) bear the greater brunt [15,16]. By June 2020, the WHO has confirmed more than 3.8 million deaths due to Covid-19 [17] pressurizing scientific organizations, institutions, and community to urgently come up with acceptable and approved vaccines. The fast rate at which these vaccines were produced coupled with various misinformation, conspiracy theories, perceived and unperceived side-effects activated public concerns and mistrust concerning whether these vaccines are harmless and/or effective [18]. About a month after the outbreak in Wuhan, China, the Federal Ministry of Health confirmed a COVID-19 case in Lagos State, Nigeria on February 27, 2020, the first case to be reported in the country since the beginning of the outbreak [19]. The Health Systems of most high-income, not to mention low-income and developing countries such as Nigeria were overwhelmed, eliciting individual, governmental, organizational, social, and medical responses. Amid the fear of the vaccine, its low supply in many parts of the world, and the persisting risk of COVID-19, interest in alternative remedies, such as diets and dietary supplements (DHS), that may enhance the immune function and reduce the risk of inflammation, remains high [20]. Calder remarked that an adequate nourishing intake may play critical role in sustaining over-all health of an individual by monitoring chronic contagious infections [21] and Kumar et al. stated that a diet rich in micronutrients could be useful in several contagious diseases [22]. DHS include all functional foods, vitamins, minerals, herbal products, essential oils, and other dietary supplements that are consumed orally to supplement the usual diet [23]. Use of supplements vary from country to country in Europe. For instance, in Germany and Denmark the prevalence is (43% and 59% of the adult population respectively) but is less in Ireland and Spain (23% and 9% respectively) while documents show that females take supplements more than males [[24], [25], [26], [27], [28]]. Data on use of dietary supplements in Africa is scarce, more especially during the recent Covid-19 pandemic. Prior to the emergence of Covid-19 pandemic, Aryeetey and Tamakloe [29] reported that multivitamin and herbal supplements are consumed regularly by elderly Ghanaians while Aina and Ojedokun [30] reported that vitamins are the common dietary supplements used by medical students in a Nigerian University. A global study however reported that interest in immune-modulating consumables increased during the COVID-19 outbreak [31]. This present study sought to assess perception, knowledge, attitude, and use of dietary supplements among Nigerians during COVID-19 infection with the specific objective to document the prevalence of dietary supplements in Nigeria during COVID-19 pandemic and the source of information on the use of these dietary supplements.

## 2 Materials and methods

### 2.1 Study design

To explore the DHS knowledge, attitude, and practices among Nigerians during the COVID-19 pandemic, a cross-sectional survey, in the form of a questionnaire was designed, and administered to adults aged over 18 years. All the study subjects were adult, male and female Nigerians able to read and understand English and were resident in Nigeria when the survey was conducted. The study subjects were males and females from various ethnic groups in the country with varying levels of educational background, employment status and marital status. The questionnaires were used to collect information on socio-demographic characteristics (including age, sex, educational status, employment status, and marital status), health status, knowledge, attitude, and behavioral pattern on the use of DHS.

The survey was conducted from April to October 2021 in two modalities: Google form and hard-copy questionnaire that was developed at the Nigerian Institute of Medical Research (NIMR) which is the primal institute of the Federal Government of Nigeria for research into health of the people. It contains many departments such as Microbiology, Public Health, Biochemistry, Human Virology and Clinical Trials. The questionnaire used in this survey was designed at the Nutrition sub-unit at the Department of Biochemistry. Due to strict restriction of movement, the survey was conducted only in Lagos State. The Google form questionnaire was sent to all the six geo-political zones of the country and the Federal Capital Territory, Abuja. However, only 206 responded with completed questionnaire. Possible reasons for non-response were (i) time limit of one week that was given (ii) the survey took place during the pandemic during which people were stressed because of lock-down, social distance and other restrictions (iii) many questionnaires were circulating online during the pandemic and (iv) response fatigue on the part of the study subjects. Because the response rate from Google form was poor, hard copies of the questionnaire were produced and administered in various communities in southwest geo-political zone which contains various ethnic groups. For this purpose, five questionnaire administrators were trained to retrieve responses from the study subjects. Face-to-face interviews were completed by trained field assistants who took all Covid-19 precautions such as wearing face mask, using hand sanitizers, maintaining social distance. Each questionnaire and material for writing were sanitized before and after use. Respondents were given face masks to wear and had their hands sanitized as well. The very few respondents who could not read nor write had the questionnaire read to them. Participants' unique identifier, as well as the intention of the survey, the process and the duration of time needed to finish each questionnaire were specified in the early part of each questionnaire. As part of the document submitted for ethical clearance, informed consent, concealment of personal identity, confidentiality and that study participants were free to decline at any point in the filling of the questionnaire were clearly stated and were read to study subjects before the survey started. Those who filled the form online had to accept to participate after reading and agreeing to terms and conditions of the study before the questionnaire could be accessed electronically. Otherwise, they had no access to the questionnaire. The consumption of any dietary and/or herbal supplement was evaluated by recollection during the Covid-19 pandemic. For the purpose of this study, dietary and/or herbal supplements were defined as any ingredient (except tobacco products) that are consumed as nutrients or micronutrients to prevent and treat Covid-19 infection. Information on the consumption pattern.

The study protocol, the questionnaire and the consent form were reviewed and approved by the Research and Ethics Committee at the Nigerian Institute of Medical Research (IRB/20/039).

### 2.2 Statistical analysis of data

Data was first extracted from Google Form as a Microsoft Excel Spreadsheet (Microsoft Corporation) and concatenated with data from hard copy questionnaire. The completed data in the Excel spreadsheet was then cleaned, coded, and exported into NCSS v2022 (Kaysville, Utah, USA) statistical software for Windows before it was analyzed, using appropriate commands. Descriptive statistical analyses concerning frequency and percentage distribution were performed for all demographic variable and the results of these were specified as numbers (n) and percentages (%). Continuous variables were reported as mean (±standard deviation [SD]) for normally distributed variables. Pearson correlation (χ^2^) with Odd Ratio (OR) at 95% Confidence Interval (CI) were carried out to determine the relationship between categorical variables of interest. Binary logistic regressions were conducted to describe the connection between sociodemographic characteristics and DHS practices during COVID-19 pandemic with consumption of DHS as dependent variable, while the sociodemographic characteristics were listed as independent variables. Results of the logistic regression models were expressed as Crude and Adjusted Odds Ratios (OR) with 95% Confidence Interval (CI). *P*-values <0.05 were considered statistically significant. Results were rendered as Tables, Graphs and Figures.

## 3 Results

### 3.1 Study participants' sociodemographic profile Table 1:

**Fig. 1:**
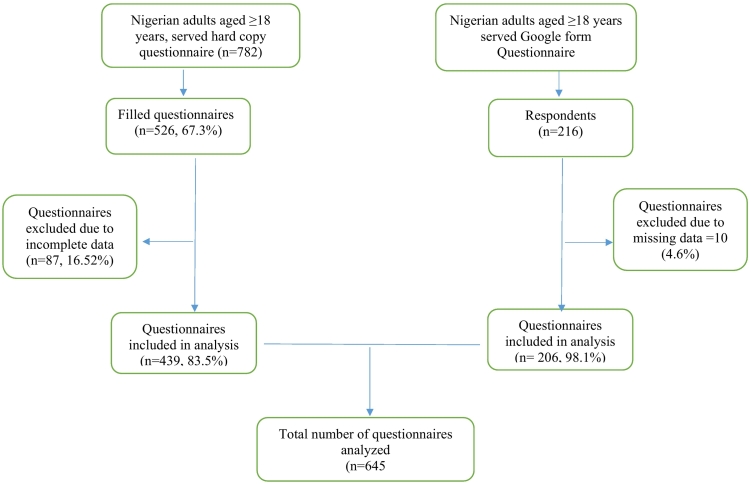
Flowchart of recruitment of study participants.

The flow chart which illustrates participant's recruitment into the study is shown in Fig. 1. Of the 782 questionnaires served, 726 (92.8%) were filled out of which 81 (11.2%) were excluded due to incomplete data, leaving 645 questionnaires that were finally analyzed. The mean (±sd) age (years) of the 645 respondents (males: 287 (44.5%); females: 358 (55.5%)) was 44.3 (12.4) with males being significantly older than females (*t*-test = 4.1, *P*-value = 0.00005). The highest (28.5%) and lowest (0.6%) proportions of the respondents were aged 41–50 years and ≤ 20 years respectively. Majority of the respondents reported being married (423, 65.6%), having tertiary educational status (436, 67.6%), employed (405, 62.8%), and of Christianity religious affiliation (Table 1). Pooled analyses show significant variation in the distributions of age groups (χ^2^ = 17.9, *P*-value = 0.006), marital status (χ^2^ = 14.9, P-value = 0.002), educational status (χ^2^ = 14.3, P-value = 0.002), occupational status (χ^2^ = 12.2, P-value = 0.002) and religious affiliation (χ^2^ = 4.6, P-value = 0.03).

**Table 1:** Socio-demographic characteristics of respondents.

Variable	Sub-variable	Statistics	All	Male	Female	χ^2^	*P*-value
Age (y)	All	Freq. (%)	645 (100.0)	287 (44.5)	358 (55.5)	–	–
		Mean (±sd)	44.3 (12.4)	46.6 (12.7)⁎	42.6 (12.0)⁎		
		Min./Max	17/80	20/80	17/78		
	≤20	Freq. (%)	4 (0.6)	2 (0.7)	2 (0.6)	17.9	0.006
	21–30		94 (14.6)	33 (11.5)	61 (17.0)		
	31–40		173 (26.8)	67 (23.3)	106 (29.6)		
	41–50		184 (28.5)	78 (27.2)	106 (29.6)		
	51–60		131 (20.3)	72 (25.1)	59 (16.5)		
	61–70		43 (6.7)	24 (8.4)	19 (5.3)		
	≥70		16 (2.5)	11 (3.8)	5 (1.4)		
Marital status	Single		140 (21.7)	50 (17.4)	90 (25.1)	14.9	0.002
	Married		423 (65.6)	209 (72.8)	214 (59.8)		
	Divorced		28 (4.3)	6 (2.1)	22 (6.2)		
	Widow/er		54 (8.4)	22 (7.7)	32 (8.9)		
Educational status	Non formal		3 (0.5)	3 (1.0)	0 (0.0)	14.3	0.002
	Primary		36 (5.6)	25 (8.7)	11 (3.1)		
	Secondary		170 (26.4)	68 (23.7)	102 (28.5)		
	Tertiary		436 (67.6)	191 (66.6)	245 (68.4)		
Occupational status	Employed		405 (62.8)	176 (61.3)	229 (64.0)	12.2	0.02
	Not employed		195 (30.2)	82 (28.6)	113 (31.5)		
	Trader		28 (4.3)	16 (5.6)	12 (3.3)		
	Retired		14 (2.2)	12 (4.2)	2 (0.6)		
	Student		3 (0.5)	1 (0.3)	2 (0.6)		
Religion	Christianity		564 (87.4)	242 (84.3)	322 (89.9)	4.6	0.03
	Islam		81 (12.6)	45 (15.7)	36 (10.1)		

⁎ t-test = 4.1, P-value = 0.00005 Male respondents were significantly older than female respondents. Pooled analyses show significant variation in the distributions of age groups (χ^2^ = 17.9, P-value = 0.006), marital status (χ^2^ = 14.9, P-value = 0.002), educational status (χ^2^ = 14.3, P-value = 0.002), occupational status (χ^2^ = 12.2, P-value = 0.002) and religious affiliation (χ^2^ = 4.6, P-value = 0.03).

### 3.2 Distribution of social habits, formal exercise, and dietary pattern of study participants by age and gender Table 2:

The percent distribution of the type of diet, smoking and alcohol consumption habits and frequency of formal exercise relative to gender and age group is as indicated in Table 2. In all 272, (42.2%) of respondents consume non-vegetarian diet, more among males (129, 45.0%) and among those aged >70 years. While a vast majority (97.2%) never smoked, especially women (99.2%) and those ≤20 years (100.0%), a lesser proportion (66.7%) did not take alcohol, mostly women (74.9%) and those ≤20 years (100.0%). However, about 50% respondents engage in formal exercise occasionally, mostly women (50.7%) and those ≤20 years (75.0%). Furthermore, the prevalence of vegetarians among study participants was approximately 12% while that of non-vegetarians was 42.3%.

**Table 2:** Distribution of social habits, exercise, and dietary pattern of respondents by age and by gender.

Variable	Category	All	Gender		Age group						
			Male	Female	≤20	21–30	31–40	41–50	51–60	61–70	>70
What type of diet do you take	Non-vegetarian	272 (42.2)	129 (45.0)	143 (39.9)	0 (0.0)	42 (44.7)	74 (42.8)	70 (38.0)	59 (45.0)	17 (38.5)	10 (62.5)
	Vegetarian	77 (11.9)	33 (11.5)	44 (12.3)	2 (50.0)	12 (12.8)	24 (13.9)	19 (10.3)	13 (9.9)	5 (11.6)	2 (12.5)
	Both	235 (36.4)	95 (33.1)	140 (39.1)	1 (25.0)	26 (27.7)	56 (32.4)	82 (44.6)	49 (37.4)	18 (41.9)	3 (18.7)
	Others	11 (1.7)	6 (2.1)	5 (1.4)	0 (0.0)	2 (2.1)	3 (1.7)	3 (1.6)	2 (1.5)	0 (0.0)	1 (6.3)
	Don't know	50 (7.8)	24 (8.3)	26 (7.3)	1 (25.0)	12 (12.8)	16 (9.2)	10 (5.4)	8 (6.1)	3 (7.0)	0 (0.0)
How often do you smoke cigarette	Never smoke	625 (97.2)	272 (94.8)	353 (99.2)	4 (100.0)	90 (95.7)	167 (97.1)	181 (98.4)	126 (96.9)	42 (97.7)	15 (93.8)
	Occasionally	12 (1.9)	10 (3.5)	2 (0.5)	0 (0.0)	3 (3.2)	3 (1.7)	2 (1.1)	3 (23)	1 (2.3)	0 (0.0)
	2–3 times/week	6 (0.9)	2 (1.7)	1 (0.3)	0 (0.0)	1 (1.1)	2 (1.2)	1 (0.5)	1 (0.8)	0 (0.0)	1 (6.2)
How often do you drink alcohol	Don't drink	430 (66.7)	162 (56.4)	268 (74.9)	4 (100.0)	58 (61.7)	105 (60.7)	126 (66.5)	94 (71.8)	29 (67.4)	14 (87.6)
	Occasionally	184 (28.5)	102 (35.5)	82 (22.9)	0 (0.0)	31 (33.0)	54 (31.2)	53 (28.8)	31 (23.7)	14 (32.6)	1 (6.2)
	Rarely	3 (0.5)	0 (0.0)	3 (0.8)	0 (0.0)	0 (0.0)	3 (1.7)	0 (0.0)	0 (0.0)	0 (0.0)	0 (0.0)
	2–3 times/week	22 (3.4)	19 (6.6)	3 (0.8)	0 (0.0)	3 (3.2)	9 (5.2)	5 (2.7)	4 (3.0)	0 (0.0)	1 (6.2)
	Once in 3 months	2 (0.3)	0 (0.0)	2 (0.6)	0 (0.0)	2 (2.1)	0 (0.0)	0 (0.0)	0 (0.0)	0 (0.0)	0 (0.0)
	Daily	4 (0.6)	4 (1.4)	0 (0.0)	0 (0.0)	0 (0.0)	2 (1.2)	0 (0.0)	2 (1.5)	0 (0.0)	0 (0.0)
How often do you engage in formal exercise	Don't exercise	111 (17.2)	54 (18.8)	57 (15.9)	1 (25.0)	15 (16.0)	25 (14.4)	34 (18.4)	20 (15.3)	13 (30.2)	3 (18.7)
	Occasionally	315 (48.8)	134 (46.7)	181 (50.6)	3 (75.0)	40 (42.5)	87 (50.3)	96 (52.2)	68 (51.9)	16 (37.2)	5 (31.3)
	2–3 times/week	98 (15.2)	45 (15.7)	53 (14.8)	0 (0.0)	13 (13.8)	33 (19.1)	25 (13.6)	18 (13.7)	6 (14.0)	3 (18.7)
	Daily	120 (18.6)	53 (18.5)	67 (18.7)	0 (0.0)	26 (27.7)	28 (16.2)	28 (15.2)	25 (19.1)	8 (18.6)	5 (31.3)
	Once a week	1 (0.2)	1 (0.3)	0 (0.0)	0 (0.0)	0 (0.0)	0 (0.0)	1 (0.5)	0 (0.0)	0 (0.0)	0 (0.0)

### 3.3 Knowledge of dietary supplements by sociodemographic characteristics of respondents Table 3:

Majority (425, 65.9%) of the study participants, mostly females (240, 56.5%), those aged 21–50 years (122, 28.7%), those with tertiary education (309, 72.7%), employed (270, 63.5%) and married individuals (276, 65.0%), agreed that dietary supplements are necessary during infectious disease outbreak such as Covid-19. In a pooled analysis, educational status strongly influenced this assertion by the (χ^2^ = 30.1, *P*-value<0.00001). In assessing study participants' awareness on whether supplements can be used exclusively to treat Covid-19, only 74 (11.5%) answered in the affirmative, especially females (44 (59.5%) and surprisingly those with tertiary education. A pooled analysis shows that age group (χ^2^ = 28.9, *P*-value = 0.004), occupational status (χ^2^ = 23.1, P-value = 0.003) and marital status (χ^2^ = 14.3, P-value = 0.03) influenced the response that Supplements can be used alone to treat COVID-19. However, majority (395, 61.2%) of the study participants, mostly females (203, 52.9%), those aged 41–50 years (114, 28.9%), those with tertiary education (304, 77.0%), those employed (257, 65.1%) and married individuals (256, 64.8%) agreed that supplements can be used in conjunction with other drugs to treat Covid-19. Both educational status (χ^2^ = 52.2, *P*-value<0.00001) and occupational status (χ^2^ = 27.7, P-value = 0.0005) influenced this response.

**Table 3:** Knowledge of dietary supplements by socio-demographic characteristics of respondents.

Variable	Category	Dietary supplements are necessary during infectious disease outbreak such as COVID-19			Supplements can be used exclusively to treat COVID-19			Supplements can be used in conjunction with other drugs to treat COVID-19		
		Yes (*n* = 425, 65.9%)	No (*n* = 58, 9.0%)	Don't know (*n* = 162, 25.1%)	Yes (*n* = 74, 11.5%)	No (*n* = 363, 56.3%)	Don't know (*n* = 208, 32.2%)	Yes (*n* = 395, 61.2%)	No (*n* = 95, 14.7%)	Don't know (*n* = 155, 24.0%)
Sex	Male	185 (43.5)	24 (41.4)	78 (58.2)	30 (40.5)	157 (43.2)	100 (48.0)	186 (47.1)	33 (34.7)	68 (43.9)
	Female	240 (56.5)	34 (58.6)	84 (51.8)	44 (59.5)	206 (56.8)	108 (52.0)	209 (52.9)	62 (65.3)	87 (56.1)
χ^2^ (P-value)		0.8 (0.67)			1.8 (0.41)			4.8 (0.09)		
Age group (yrs.)	≤20	3 (0.7)	0 (0.0)	1 (0.6)	1 (1.3)	3 (0.8)	0 (0.0)	2 (0.5)	1 (1.0)	1 (0.6)
	21–30	67 (15.8)	7 (12.1)	20 (12.4)	13 (17.6)	60 (16.5)	21 (10.1)	67 (17.0)	11 (11.6)	16 (10.3)
	31–40	109 (25.6)	15 (25.9)	49 (30.3)	23 (31.1)	97 (26.7)	53 (25.5)	105 (26.6)	21 (22.1)	47 (30.3)
	41–50	122 (28.7)	19 (32.8)	43 (26.5)	23 (31.1)	94 (26.0)	67 (32.2)	114 (28.9)	27 (28.4)	43 (27.7)
	51–60	94 (22.1)	7 (12.1)	30 (18.5)	11 (14.9)	80 (22.0)	40 (19.2)	74 (18.7)	24 (25.3)	33 (21.3)
	61–70	22 (5.2)	7 (12.1)	14 (8.6)	1 (1.3)	27 (7.4)	15 (7.2)	26 (6.6)	9 (9.5)	8 (5.2)
	>70	8 (1.9)	3 (5.2)	5 (3.1)	2 (2.7)	2 (0.6)	12 (5.8)	7 (1.7)	2 (2.1)	7 (4.5)
χ^2^ (P-value)		18.3 (0.11)			28.9 (0.004)			12.8 (0.39)		
Educational status	Non formal	0 (0.0)	0 (0.0)	3 (1.9)	0 (0.0)	0 (0.0)	3 (1.4)	0 (0.0)	0 (0.0)	3 (1.9)
	Primary	16 (3.8)	2 (3.5)	18 (11.1)	6 (8.1)	18 (5.0)	12 (5.8)	16 (4.0)	11 (11.6)	9 (5.8)
	Secondary	100 (23.5)	18 (31.0)	52 (32.1)	24 (32.4)	88 (24.2)	58 (27.9)	75 (19.0)	38 (40.0)	57 (36.8)
	Tertiary	309 (72.7)	38 (65.5)	89 (54.9)	44 (59.5)	257 (70.8)	135 (64.9)	304 (77.0)	46 (48.2)	86 (55.5)
χ^2^ (P-value)		30.1 (<0.00001)			10.8 (0.10)			52.2 (<0.00001)		
Occupational status	Employed	270 (63.5)	30 (51.7)	105 (64.8)	48 (64.9)	231 (63.4)	126 (60.6)	257 (65.1)	52 (54.7)	96 (61.9)
	Not employed	132 (31.1)	20 (34.5)	43 (26.5)	25 (33.8)	110 (30.3)	60 (28.8)	119 (30.1)	26 (27.4)	50 (32.3)
	Trader	15 (3.5)	5 (8.6)	8 (4.9)	1 (1.3)	19 (5.2)	8 (3.8)	12 (3.0)	13 (13.7)	3 (1.9)
	Retired	7 (1.7)	3 (5.2)	4 (2.5)	0 (0.0)	2 (0.6)	12 (5.8)	6 (1.5)	3 (3.2)	5 (3.2)
	Student	1 (0.2)	0 (0.0)	2 (1.2)	0 (0.0)	1 (0.3)	2 (1.0)	1 (0.3)	1 (1.0)	1 (0.6)
χ^2^ (P-value)		11.5 (0.18)			23.1 (0.003)			27.7 (0.0005)		
Marital Status	Single	97 (22.8)	10 (17.2)	33 (20.4)	24 (32.4)	86 (23.7)	30 (14.4)	97 (24.6)	15 (15.8)	28 (18.1)
	Married	276 (65.0)	37 (63.8)	110 (67.9)	40 (54.1)	236 (65.0)	147 (70.7)	256 (64.8)	64 (67.3)	103 (66.4
	Separated	20 (4.7)	5 (8.6)	3 (1.9)	3 (4.0)	16 (4.4)	9 (4.3)	18 (4.5)	3 (3.2)	7 (4.5)
	Widow	38 (7.5)	6 (10.3)	16 (9.9)	7 (9.5)	25 (6.9)	22 (10.6)	24 (6.1)	13 (13.7)	17 (11.0)
χ^2^ (P-value)		6.2 (0.40)			14.3 (0.03)			11.4 (0.08)		

WARNING: At least one cell had an expected value less than 5.

### 3.4 Dietary supplements (DS) known by respondents Table 4, Fig. 2a:

Study participants were asked about their knowledge of DHS to prevent or treat Covid-19 during the pandemic. Of all the vitamins indicated, Vitamin C (Ascorbic acid) is the most known (70%) and Vitamin A is the least known (0.3%) DS by all the participants across sociodemographic characteristics. Approximately half (50.2%) of the study subjects, more than a third (37.8%) and less than a quarter (22.7%) were aware that Folic acid, vitamin D and vitamin E are DS. Also, relatively low proportions of study participants recognized selenium (11.8%), iron (30.5%), zinc (29.5%) as DS though a higher proportion (40.2%) recognized calcium as such. Herbal dietary supplements mentioned as known by the study participants included Garlic (46.5%), Ginger (44.7%), Tumeric (36.3%), Moringa (40.0%) and Ginseng (26.3%). Citrus fruit as a DS was recognized by fewer (6.5%) study participants and only 1.6% referred to herbal tea as DHS.

**Table 4:** Frequency distribution of dietary supplements reported as known by respondents.

Supplement	Types of dietary supplements mentioned as known															
	Age group							Educational status				Occupational status				
	≤20	21–30	31–40	41–50	51–60	61–70	>70	None	1º	2º	3º	Employed	Not Employed	Trader	Retiree	Student
	Types of Supplements mentioned as known															
Selenium	0 (0.0)	5 (5.3)	27 (15.6)	26 (14.1)	13 (9.9)	5 (11.6)	0 (0.0)	0 (0.0)	1 (2.8)	14 (8.2)	61 (14.0)	56 (13.8)	20 (10.3)	0 (0.0)	0 (0.0)	0 (0.0)
Vitamin A	0 (0.0)	0 (0.0)	1 (0.6)	0 (0.0)	1 (0.8)	0 (0.0)	0 (0.0)	0 (0.0)	1 (2.8)	1 (0.6)	0 (0.0)	1 (0.3)	0 (0.0)	1 (3.6)	0 (0.0)	0 (0.0)
Vitamin Bco	2 (50.0)	51 (54.3)	90 (52.0)	88 (48.1)	68 (51.9)	14 (32.6)	2 (12.5)	3 (100.0)	15 (41.7)	66 (38.8)	231 (53.0)	205 (50.6)	105 (53.9)	3 (10.7)	1 (7.1)	1 (33.3)
Vitamin C	3 (75.0)	67 (71.3)	122 (70.5)	132 (71.7)	94 (71.8)	27 (62.8)	12 (75.0)	3 (100.0)	21 (58.3)	114 (67.1)	319 (73.2)	295 (72.8)	144 (73.9)	9 (32.1)	6 (42.9)	3 (100.0)
Vitamin D	1 (25.0)	42 (44.7)	81 (46.8)	61 (33.2)	41 (31.3)	43 (41.9)	0 (0.0)	3 (100.0)	3 (8.3)	40 (23.5)	198 (45.4)	166 (41.0)	75 (38.5)	3 (10.7)	0 (0.0)	3 (100.0)
Vitamin E	1 (25.0)	25 (26.9)	42 (24.3	39 (21.2)	30 (22.9)	8 (18.6)	1 (6.3)	0 (0.0)	2 (5.6)	32 (18.8)	112 (25.8)	97 (24.0)	48 (24.6)	1 (3.6)	0 (0.0)	0 (0.0)
Folic Acid	2 (50.0)	55 (58.5)	93 (53.8)	92 (50.0)	61 (46.6)	19 (44.2)	2 (12.5)	3 (100.0)	12 (33.3)	65 (38.2)	244 (56.0)	221 (54.6)	97 (49.7)	5 (17.9)	0 (0.0)	1 (33.3)
Iron	1 (25.0)	37 (39.4)	59 (34.1)	55 (29.9)	32 (24.4)	11 (25.6)	2 (12.5)	3 (100.0)	3 (8.3)	30 (17.7)	161 (36.9)	136 (33.6)	58 (29.7)	1 (3.6)	2 (14.3)	0 (0.0)
Calcium	2 (50.0)	44 (46.8)	79 (45.7)	66 (35.9)	45 (34.4)	18 (41.9)	5 (31.3)	3 (100.0)	6 (16.7)	46 (27.1)	204 (46.8)	172 (42.5)	81 (41.5)	3 (10.7)	3 (21.4)	0 (0.0)
Zinc	1 (25.0)	32 (34.0)	51 (29.5)	51 (27.7)	39 (29.8)	16 (37.2)	0 (0.0)	3 (100.0)	3 (8.3)	32 (18.8)	152 (34.9)	133 (32.8)	54 (27.7)	3 (10.7)	0 (0.0)	0 (0.0)
Multivite	2 (50.0)	47 (50.0)	74 (42.8)	81 (44.0)	56 (42.8)	20 (46.5)	4 (25.0)	3 (100.0)	9 (25.0)	62 (36.5)	206 (47.3)	191 (47.2)	79 (40.5)	7 (25.0)	4 (27.6)	3 (100.0)
Cellgevity	1 (25.0)	15 (16.0)	23 (13.3)	10 (5.4)	15 (11.5)	5 (11.6)	0 (0.0)	0 (0.0)	4 (11.1)	12 (7.1)	53 (12.2)	45 (11.1)	20 (10.3)	3 (10.7)	1 (7.1)	0 (0.0)

	Types of herbal dietary supplements mentioned as known															
Garlic	2 (50.0)	54 (57.5)	72 (41.6)	86 (46.7)	58 (44.3)	23 (53.5)	5 (31.3)	0 (0.0)	9 (25.0)	65 (38.2)	226 (51.8)	188 (46.4)	104 (53.3)	4 (14.3)	3 (21.4)	1 (33.3)
Ginger	3 (75.0)	54 (57.5)	71 (41.0)	84 (45.7)	50 (38.2)	20 (46.5)	6 (37.5)	3 (100.0)	9 (25.0)	55 (32.4)	221 (50.7)	186 (45.9)	91 (46.7)	7 (25.0)	3 (21.4)	1 (33.3)
Tumeric	1 (25.0)	36 (38.3)	63 (36.2)	64 (34.8)	48 (36.6)	17 (39.5)	5 (31.3)	0 (0.0)	7 (19.4)	45 (26.5)	182 (41.7)	152 (37.5)	75 (38.5)	3 (10.7)	3 (21.4)	1 (33.3)
Moringa	2 (50.0)	47 (50.0)	64 (37.0)	73 (39.7)	48 (36.6)	20 (46.5)	4 (25.0)	3 (100.0)	9 (25.0)	47 (27.7)	199 (45.6)	164 (40.5)	87 (44.6)	3 (10.7)	3 (21.4)	1 (33.3)
Ginseng	0 (0.0)	25 (26.9)	55 (31.8)	46 (25.3)	28 (21.4)	14 (32.6)	1 (6.3)	0 (0.0)	4 (11.4)	26 (15.3)	139 (32.0)	115 (28.5)	53 (27.3)	1 (3.6)	0 (0.0)	0 (0.0)
Herbal tea	0 (0.0)	1 (1.1)	2 (1.2)	5 (2.7)	2 (1.5)	0 (0.0)	0 (0.0)	0 (0.0)	3 (8.3)	6 (3.5)	1 (0.2)	9 (2.2)	1 (0.5)	0 (0.0)	0 (0.0)	0 (0.0)

	Type of fruit supplement mentioned as known															
Citrus	0 (0.0)	14 (14.9)	22 (12.7)	7 (3.8)	2 (1.5)	0 (0.0)	0 (0.0)	0 (0.0)	0 (0.0)	0 (0.0)	45 (10.3)	28 (6.9)	16 (8.2)	1 (3.6)	0 (0.0)	0 (0.0)

**Fig. 2:**
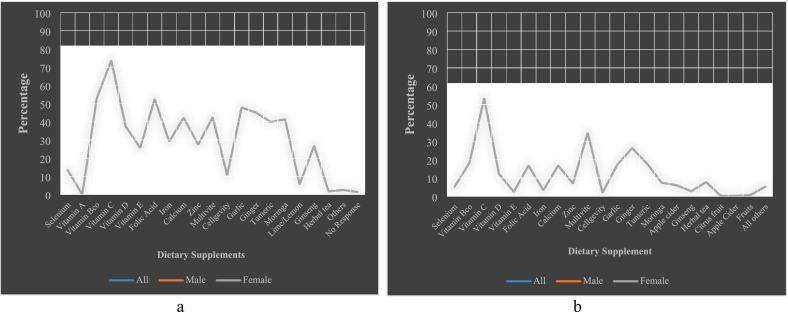
(a). Distribution of dietary/herbal/citrus fruit supplements mentioned as known (a) and consumed (b) by respondents during the Covid-19 period relative to gender. (b). refers: *Females were 1.3 times more likely to take vitamin C (χ*^*2*^*= 2.5, P-value = 0.11, OR = 1.3, 95% CI = 0.94, 1.75) and 1.1 times more likely to take multivitamin (χ*^*2*^*= 0.4, P-value = 0.55, OR = 1.1, 95% CI = 0.80, 1.53) than males, while males were 1.6 times more likely to take vitamin D more than females (χ*^*2*^*= 3.6, P-value = 0.06, OR = 1.6, 95% CI = 0.99, 2.49). While females were approximately twice as likely to take Selenium (χ*^*2*^*= 2.5, P-value = 0.11, OR = 1.95, 95% CI = 0.84, 4.53) and were 1.2 times more likely to take Calcium (χ*^*2*^*= 0.7, P-value = 0.39, OR = 1.2, 95% CI = 0.78, 1.86) than males, males were about 2½ more likely to consume Iron (χ*^*2*^*= 3.5, P-value = 0.06, OR = 2.4, 95% CI = 0.94, 6.04) and approximately twice as likely to consume Zinc (χ*^*2*^*= 5.8, P-value = 0.02, OR = 1.9, 95% CI = 1.12, 3.27) than females.*

### 3.5 Consumption and frequency of use of DHS during Covid-19 pandemic Table 5.

In all, 571 (88.5%) of the study participants took dietary supplements during the Covid-19 pandemic, especially females (324, 56.7%), those aged 41–50 years (163, 28.6%), those with tertiary education (392, 68.7%), those employed (358, 62.7%) and those married (379, 66.4%). Females were 1.5 times more likely to take DHS during Covid-19 pandemic than males (χ^2^ = 3.1, *P*-value = 0.08, OR = 1.5, 95% CI = 0.95, 2.47). A marginally significant distribution was observed in the consumption of DHS relative to age groups. Of those who took DHS more during Covid-19 pandemic, 235 (41.2%) took it daily, 167 (29.3%) took it occasionally, 83 (14.5%) on alternate days, 40 (7.0%) once a week, 35 (5.8%) twice a week and only 13 (2.3%) took it when they remember or prompted by other. In pooled analyses, significant differences were observed in the proportions of frequency of consumption of DHS by Age group (χ^2^ = 51.3, *P*-value = 0.009) and by occupational status (χ^2^ = 54.9, P-value = 0.000001).

**Table 5:** Consumption and frequency of use of dietary supplements during Covid-19 pandemic in Nigeria.

Variable	Category	Do you take dietary supplements more during this Covid-19		If yes, how often do you take dietary supplements					
		Yes	No	Occasionally	Alternate days	Daily	Once a week	Twice a week	Others
All		571 (88.5)	74 (11.5)	167 (29.3)	83 (14.5)	235 (41.2)	40 (7.0)	33 (5.8)	13 (2.3)
Sex	Male	247 (43.3)	40 (54.1)	79 (47.3)	35 (42.2)	95 (40.4)	22 (55.0)	12 36.4()	4 (30.8)
	Female	324 (56.7)	34 (45.9)	91 (52.7)	48 (57.8)	140 (59.6)	18 (45.0)	21 (63.6)	9 (69.2)
χ^2^ (P-value)		3.1 (0.08)		5.2 (0.39)					
OR (95% CI)	Male vs Female	0.7 (0.40, 1.05)		–					
	Female vs Male	1.5 (0.95, 2.47)		–					
Age group (yrs.)	≤20	3 (0.5)	1 (1.4)	0 (0.0)	1 (1.2)	2 (0.9)	0 (0.0)	0 (0.0)	0 (0.0)
	21–30	74 (13.0)	20 (27.0)	30 (18.0)	11 (13.3)	19 (8.1)	5 (12.5)	7 (21.2)	2 (15.4)
	31–40	157 (27.5)	16 (21.6)	45 (27.0)	29 (34.9)	60 (25.5)	16 (40.0)	3 (9.1)	4 (30.8)
	41–50	163 (28.6)	21 (28.4)	52 (31.1)	20 (24.1)	71 (30.2)	9 (22.5)	8 (24.2)	3 (23.1)
	51–60	119 (20.8)	12 (16.2)	29 (17.4)	16 (19.3)	60 (25.5)	4 (10.0)	7 (21.2)	3 (23.1)
	61–70	40 (7.0)	3 (4.1)	6 (3.6)	3 (3.6)	21 (8.9)	5 (12.5)	4 (12.1)	1 (7.7)
	>70	15 (2.6)	1 (1.3)	5 (3.0)	3 (3.6)	2 (0.9)	1 (2.5)	4 (12.1)	0 (0.0)
χ^2^ (P-value)		12.4 (0.05)		51.4 (0.009)					
Educational status	Non formal	3 (0.5)	0 (0.0)	3 (1.8)	0 (0.0)	0 (0.0)	0 (0.0)	0 (0.0)	0 (0.0)
	Primary	28 (4.9)	8 (10.8)	3 (1.8)	5 (6.0)	16 (6.8)	3 (7.5)	1 (3.0)	0 (0.0)
	Secondary	148 (25.9)	22 (29.7)	41 (24.60)	15 (18.1)	62 (26.4)	12 (30.0)	12 (36.4)	6 (46.2)
	Tertiary	392 (68.7)	44 (59.5)	120 (71.9)	63 (75.9)	157 (66.8)	25 (62.5)	20 (60.6)	7 (53.8)
χ^2^ (P-value)		5.7 (0.13)		21.7 (0.12)					
Occupational status	Employed	358 (62.7)	47 (63.5)	103 (61.7)	58 (69.9)	140 (59.6)	25 (62.5)	23 (66.7)	10 (76.9)
	Not employed	172 (30.1)	23 (31.1)	57 (34.1)	21 (25.3)	78 (33.2)	12 (30.0)	3 (9.1)	1 (7.7)
	Trader	25 (4.4)	3 (4.0)	4 (2.4)	3 (3.6)	12 (5.1)	2 (5.0)	3 (9.1)	1 (7.7)
	Retired	13 (2.3)	1 (1.4)	3 (1.8)	1 (1.2)	3 (1.3)	1 (2.5)	5 (15.2)	0 (0.0)
	Student	3 (0.5)	0 (0.0)	0 (0.0)	0 (0.0)	2 (0.9)	0 (0.0)	0 (0.0)	1 (7.7)
χ^2^ (P-value)		0.7 (0.95)		54.9 (0.000001)					
Marital status	Single	117 (20.5)	23 (31.1)	41 (24.6)	14 (16.9)	41 (17.5)	9 (22.5)	9 (27.3)	3 (23.1)
	Married	379 (66.4)	44 (59.5)	105 (62.9)	57 (68.7)	162 (68.9)	25 (62.5)	20 (60.6)	10 (76.9)
	Separated	25 (4.4)	3 (4.0)	9 (5.4)	3 (3.6)	12 (5.1)	1 (2.5)	0 (0.0)	0 (0.0)
	Widow	50 (8.8)	4 (5.4)	12 (7.2)	9 (10.8)	20 (8.5)	5 (12.5)	4 (12.1)	0 (0.0)
χ^2^ (P-value)		4.7 (0.19)		11.1 (0.75)					

### 3.6 DHS consumed during Covid-19 pandemic in Nigeria Table 6, Fig. 2b:

The most consumed vitamin during Covid-19 pandemic in Nigeria was Vitamin C, taken by 346 (53.6%) of the study participants, followed by multivitamins (33.6%). Vitamin C was consumed mostly in the age group of 41–50 years (56.5%) followed closely by those in age group ≤20 years (56.1%) and 31–40 years (52.6%). Older age groups >60 years least consumed vitamin C. Stratified by educational status, those with tertiary education mostly (53.9%) consumed vitamin C while those with primary or no education consumed it least of all (38.5%). Surprisingly, the consumption of vitamin C was highest (64.3%) among singles and least (48.7%) among married individuals in the study. Lesser proportions consumed Folic acid (119, 18.5%), Vitamin D (79, 12.3%) and Vitamin E (16, 2.3%). Females were 1.3 times more likely to consume vitamin C (χ^2^ = 2.5, *P*-value = 0.11, OR = 1.3, 95% CI = 0.94, 1.75) and 1.1 times more likely to consume multivitamin (χ^2^ = 0.4, P-value = 0.55, OR = 1.1, 95% CI = 0.80, 1.53) than males, while males were 1.6 times more likely to consume vitamin D more than females (χ^2^ = 3.6, P-value = 0.06, OR = 1.6, 95% CI = 0.99, 2.49). Participants also reported consumption of Selenium (27, 4.2%), Iron (20, 3.1%), Zinc (61, 9.5%) and Calcium (101, 15.7%) to prevent/treat Covid-19. Calcium was the commonest (101, 15.7%) mineral consumed followed by Zinc (61, 9.5%), Selenium (27, 4.2%) and Iron (20, 3.1%). The highest consumption of Calcium was in the older age group of 51–60 years (26, 19.8%), >70 years (3, 18.8%) and 51–70 years (8, 18.6%) and surprisingly by those with secondary education level (29, 17.1%) and widows (9, 16.7%). While females were approximately twice as likely to take Selenium (χ^2^ = 2.5, *P*-value = 0.11, OR = 2.0, 95% CI = 0.84, 4.53) and were 1.2 times more likely to take Calcium (χ^2^ = 0.7, P-value = 0.39, OR = 1.21, 95% CI = 0.78, 1.86) than males, males were about 2½ more likely to consume Iron (χ^2^ = 3.5, P-value = 0.06, OR = 2.4, 95% CI = 0.94, 6.04) and approximately twice as likely to consume Zinc (χ^2^ = 5.8, P-value = 0.02, OR = 1.9, 95% CI = 1.12, 3.27) than females. Herbal dietary consumed by study participants to prevent/treat Covid-19 included Garlic (118, 18.3%), Ginger (167, 25.9%), Tumeric (102, 15.8%), Moringa (59, 9.2%) and Ginseng (23, 3.6%). Citrus fruit was hardly consumed (1, 0.2%) but surprisingly a higher proportion (53, 8.2%) consumed herbal tea to prevent/treat Covid-19 pandemic.

**Table 6:** Dietary supplements consumed by different categories of respondents during Covid-19 pandemic in Nigeria.

Supplement	Types of dietary supplements consumed during Covid-19 pandemic																
	Age group						Educational status			Occupational status				Marital status			
	≤30 (*n* = 98)	31–40 (*n* = 173)	41–50 (*n* = 184)	51–60 (*n* = 131)	61–70 (*n* = 43)	>70 (*n* = 16)	≤1° (*n* = 39)	2° (*n* = 170)	3° (*n* = 436)	Employed (*n* = 405)	Not-Employed (*n* = 198)	Trader (*n* = 28)	Retiree (*n* = 14)	Single (*n*-140)	Married (*n* = 423)	Divorced (*n* = 28)	Widow (54)
	Types of Supplements mentioned as taken																
Selenium	0 (0.0)	5 (2.9)	11 (6.0)	9 (6.9)	2 (4.7)	0 (0.0)	0 (0.0)	9 (5.3)	18 (4.1)	17 (4.2)	10 (5.1)	0 (0.0)	0 (0.0)	4 (2.9)	17 (4.0)	0 (0.0)	6 (11.1)
Vitamin B co	15 (15.3)	16 (9.2)	35 (19.0)	34 (26.0)	6 (14.0)	1 (6.3)	8 (20.5)	41 (24.1)	58 (13.3)	58 (14.3	48 (24.2)	1 (3.6)	0 (0.0)	18 (12.9)	71 (16.8)	4 (14.3)	14 (25.9)
Vitamin C	55 (56.1)	91 (52.6)	104 (56.5)	64 (48.9)	19 (44.2)	7 (43.8)	15 (38.5)	90 (52.9)	235 (53.9)	221 (54.6)	98 (49.5)	14 (50.0)	7 (50.0)	90 (64.3)	206 (48.7)	14 (50.0)	30 (55.6)
Vitamin D	17 (17.3)	25 (14.5)	12 (6.5)	20 (15.3)	5 (11.6)	0 (0.0)	1 (2.6)	18 (10.6)	60 (13.8)	55 (13.6)	21 (10.6)	2 (7.1)	1 (7.1)	22 (15.7)	46 (11.6)	3 (10.7)	5 (9.3)
Vitamin E	4 (4.1)	5 (2.9)	2 (1.1)	2 (1.5)	3 (7.0)	0 (0.0)	0 (0.0)	5 (2.9)	11 (2.5)	8 (2.0)	8 (4.0)	0 (0.0)	0 (0.0)	5 (3.6)	7 (1.7)	3 (10.7)	1 (1.9)
Folic Acid	16(16.3)	29 (16.8)	37 (20.1)	24 (18.3)	8 (18.6)	2 (12.5)	4 (10.3)	30 (17.6)	82 (18.8)	78 (19.3)	31 (15.7)	5 (17.9)	2 (14.3)	26 (18.6)	73 (17.3)	4 (14.3)	13 (24.1)
Iron	5 (5.1)	6 (3.5)	5 (2.7)	2 (1.5)	2 (4.7)	0 (0.0)	2 (5.1)	2 (1.2)	16 (3.7)	13 (3.2)	6 (3.0)	1 (3.6)	0 (0.0)	3 (2.1)	16 (3.8)	0 (0.0)	1 (1.9)
Calcium	16 (16.3)	23 (13.3)	25 (13.6)	26 (19.8)	8 (18.6)	3 (18.8)	5 (12.8)	29 (17.1)	67 (15.4)	59 (17.0)	23 (11.6)	6 (21.4)	3 (21.4)	20 (14.3)	68 (16.1)	4 (14.3)	9 (16.7)
Zinc	3 (3.1)	13 (7.5)	23 (12.5)	15 (11.5)	6 (14.0)	1 (6.3)	3 (7.7)	9 (5.3)	49 (11.2)	44 (10.9)	14 (7.1)	3 (10.7)	0 (0.0)	10 (7.1)	50 (11.8)	0 (0.0)	1 (1.9)
Multivitamin	33 (33.7)	61 (35.3)	58 (31.5)	48 (36.6)	16 (37.2)	1 (6.3)	11 (28.2)	57 (33.5)	149 (34.2)	143 (35.3)	58 (34.3)	4 (14.3)	2 (14.3)	45 (32.1)	144 (34.0)	11 (39.3)	17 (31.5)
Cellgevity	3 (3.1)	2 (1.2)	3 (1.6)	6 (4.6)	0 (0.0)	0 (0.0)	1 (2.6)	3 (1.8)	10 (2.3)	11 (2.7)	1 (0.5)	1 (3.6)	1 (7.1)	5 (3.6)	7 (1.7)	0 (0.0)	2 (3.7)
Others	1 (1.0)	3 (1.7)	6 (3.3)	4 (3.1)	1 (0.0)	0 (0.0)	1 (2.6)	4 (2.4)	10 (2.3)	9 (2.2)	3 (1.5)	1 (3.6)	1 (7.1)	1 (0.7)	11 (2.6)	2 (7.1)	1 (1.9)

	Types of herbal supplements mentioned as consumed																
Garlic	15 (15.3)	23 (15.3)	37 (20.1)	28 (21.4)	14 (32.6)	1 (6.3)	6 (15.4)	29 (17.1)	83 (19.0)	75 (18.5)	40 (20.2)	3 (10.7)	0 (0.0)	23 (16.4)	81 (19.1)	3 (10.7)	11 (20.4)
Ginger	28 (28.6)	42 (24.3)	51 (27.7)	29 (22.1)	15 (34.9)	2 (12.5)	7 (17.9)	38 (22.4)	122 (28.0)	103 (25.4)	59 (29.8)	4 (14.3)	1 (7.1)	41 (29.3)	104 (24.6)	5 (17.9)	17 (31.5)
Tumeric	8 (8.2)	31 (17.9)	30 (16.3)	21 (16.0)	12 (27.9)	0 (0.0)	0 (0.0)	20 (11.8)	82 (18.8)	66 (16.3)	35 (17.7)	1 (3.6)	0 (0.0)	22 (15.7)	71 (16.8)	1 (3.6)	8 (14.6)
Moringa	5 (5.1)	8 (4.6)	22 (12.0)	17 (13.0)	6 (14.0)	1 (6.2)	7 (17.9)	19 (11.2)	33 (7.6)	41 (10.1)	17 (8.6)	1 (3.6)	0 (0.0)	7 (5.0)	44 (10.4)	2 (7.1)	6 (11.1)
Ginseng	1 (1.0)	2 1.20)	12 (6.5)	6 (4.6)	2 (4.7)	0 (0.0)	1 (2.6)	6 (3.5)	16 (3.7)	12 (3.0)	9 (4.5)	2 (2.7)	0 (0.0)	1 (0.7)	20 (4.7)	1 (3.6)	1 (1.9)
Herbal tea	3 (3.1)	15 (8.7)	11 (6.0)	15 (11.5)	7 (16.3)	2 (12.5)	5 (12.8)	12 (7.1)	36 (8.3)	27 (6.7)	13 (6.6)	11 (39.3)	2 (14.3)	0 (0.0)	47 (11.1)	2 (7.1)	4 (7.4)
Others	1 (1.0)	1 (0.6)	1 (0.5)	1 (0.8)	3 (7.0)	0 (0.0)	0 (0.0)	3 (1.8)	4 (0.9)	2 (0.5)	5 (2.5)	0 (0.0)	0 (0.0)	1 (0.7)	2 (0.5)	3 (10.7)	1 (1.9)

	Types of citrus fruits mentioned as consumed																
Lime/Lemon	0 (0.0)	0 (0.0)	1 (0.5)	0 (0.0)	0 (0.0)	0 (0.0)	0 (0.0)	0 (0.0)	1 (0.2)	1 (0.2)	0 (0.0)	0 (0.0)	0 (0.0)	0 (0.0)	1 (0.2)	0 (0.0)	0 (0.0)
Apple Cider	5 (5.1)	9 (5.2)	9 (4.9)	6 (4.6)	0 (0.0)	0 (0.0)	2 (5.1)	8 (4.7)	19 (4.4)	20 (4.9)	9 (4.5)	0 (0.0)	0 (0.0)	7 (5.0)	18 (4.3)	1 (3.6)	3 (5.6)
Fruits	0 (0.0)	0 (0.0)	3 (1.6)	1 (0.8)	0 (0.0)	0 (0.0)	0 (0.0)	3 (1.8)	0 (0.0)	3 (0.7)	1 (0.5)	0 (0.0)	0 (0.0)	2 (1.4)	2 (0.5)	0 (0.0)	0 (0.0)

^⁎^Vitamin B co = Vitamin B complex.

### 3.7 Source of information on dietary and herbal supplements Fig. 3:

Study participants were asked about how they got information for the consumption of various DHS to prevent/treat Covid-19 during the pandemic. Incidentally, majority (271, 42.0%) depended on health worker for information on the use of dietary and herbal products while 13% mentioned Social media, 12% mentioned books, another 12% mentioned friend/relative/neighbor, 9% said they got the information from the internet and only 6% mention Mass media (TV/Radio) as the source of their information.

**Fig. 3:**
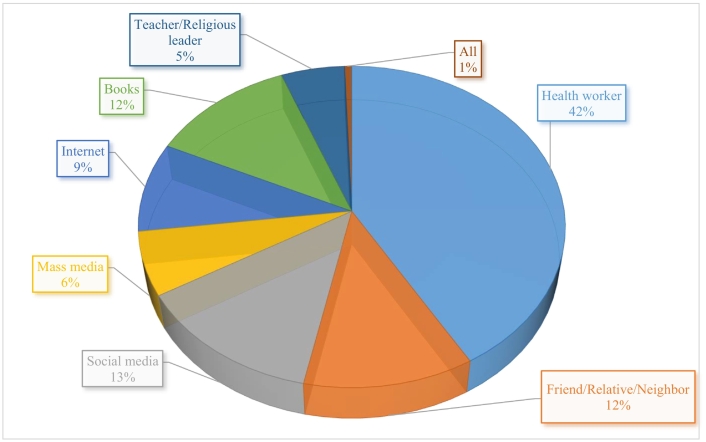
Source of information on practices related to food and dietary supplements used to prevent/treat COVID-19*. (*Percentage among the pool of answers excluding those who did not specify.)

### 3.8 Sociodemographic determinants of use of DHS to prevent/treat Covid-19 during the pandemic Table 7:

The sociodemographic determinants of DHS practices used to prevent/treat COVID-19 during the pandemic were examined using simple and multiple logistic regressions and the results are summarized in Tables 7. Increased intake of supplements was more common among those aged 61–70 years and those aged 41–50 years (vs ≤30 years), among widows (vs single) among those with secondary education (vs ≤ Primary) and among those not employed (vs employed). Increased intake of herbal products as DHS was also more common in the age-group of 61–70 years, widows, those with tertiary education and those not employed. Factors associated with increased intake of citrus fruit during Covid-19 pandemic were (i) older age of 61–70 years, (ii) widowhood, (iii) secondary level of education and (iv) not employed.

**Table 7:** Multivariate regression analysis of the association between socio-demographic characteristics and DHS practice (increased intake of supplements, herbal products and citrus fruits) during Covid-19 pandemic.

	Increased intake of supplements				Increased intake of herbal products^⁎^				Increased intake of citrus fruits			
	COR	95% CI	AOR	95% CI	COR	95% CI	AOR	95% CI	COR	95% CI	AOR	95% CI
Sex (ref: Male)												
Female	**1.0**	0.61–1.69	1.0	0.57–1.62	**1.2**	0.87–1.62	1.2	0.84–1.63	0.7	0.38–1.11	0.7	0.41–1.27
Age (ref: ≤30)												
31–40	1.0	0.42–2.19	0.9	0.32–2.32	1.1	0.65–1.76	1.3	0.70–2.33	0.9	0.29–2.84	0.6	0.16–2.36
41–50	**1.1**	0.51–2.51	1.1	0.40–2.98	1.1	0.67–1.79	1.4	0.76–2.66	2.5	0.93–6.89	1.5	0.43–5.49
51–60	0.9	0.37–2.15	0.8	0.27–2.56	1.2	0.73–2.09	1.8	0.89–3.50	2.8	0.99–7.80	1.7	0.45–6.55
61–70	**1.7**	0.60–4.85	1.7	0.47–5.79	**1.7**	0.82–3.61	2.4	0.98–5.64	**3.0**	0.87–10.49	2.5	0.54–11.17
>70	0.6	0.07–4.92	0.7	0.57–9.42	0.2	0.06–0.79	0.3	0.07–1.52	1.2	0.14–11.36	1.5	0.12–18.04
Marital status (ref: Single)												
Married	1.1	0.57–2.02	1.2	0.50–2.62	0.9	0.62–1.34	0.8	0.49–1.36	2.2	0.97–5.02	1.6	0.55–4.66
Divorced	1.1	0.29–4.04	1.0	0.22–4.11	0.9	0.41–2.07	0.7	0.29–1.82	1.5	0.29–7.44	0.9	0.15–5.35
Widowed	**1.1**	0.41–3.10	1.3	0.39–4.40	**1.1**	0.57–2.02	1.2	0.52–2.53	**2.4**	0.76–7.42	1.8	0.46–7.04
Educational status (ref: Primary)												
Secondary	**2.5**	0.55–11.03	2. 6	0.56–11.84	1.0	0.49–1.95	0.8	0.40–1.73	**0.6**	0.22–1.48	0.61	0.22–1.71
Tertiary	2.2	0.51–9.35	2.2	0.49–9.78	**1.2**	0.63–2.33	1.1	0.56–2.26	0.4	0.15–0.91	0. 5	0.17–0.19
Occupational status (ref: employed)												
Not employed	**1.0**	0.58–1.72	1.0	0.54–1.69	**1.2**	0.85–1.69	1.3	0.89–1.83	**0.8**	0.46–1.51	0.7	0.38–1.36
Trader	0.0	–	–	–	0.6	0.25–1.20	0.5	0.23–1.18	0.3	0.04–2.48	0.2	0.03–1.51
Retired	0.6	0.08–4.82	0.5	0.04–6.51	0.3	0.11–1.10	0.6	0.13–2.93	0.00	–	0.0	–

*This includes garlic, ginger, moringa, tumeric, ginseng, herbal tea: COR = Crude ODHS Ratio; AOR = Adjusted ODHS ratio.

### 3.9 Reasons for consuming dietary and herbal supplements during Covid-19 pandemic in Nigeria Fig. 4

When asked about the reasons why study respondents consumed dietary and herbal supplements during the Covid-19 pandemic in the country, majority (66.4%) responded that it was to maintain good health while lesser proportions said that they wanted to prevent ill health (26.2%), maintain adequate nutrition (18.8%) or hasten recovery from illness (9.8%). Females were 1½ times, 1.3 times and about 1.1 times more likely to use DHS to prevent weight loss (χ^2^ = 1.2, *P*-value = 0.27, OR = 1.5, 95% CI = 0.73, 3.09), to maintain adequate nutrition (χ^2^ = 1.4, *P*-value = 0.24, OR = 1.3, 95% CI = 0.85, 1.91) and to maintain good health (χ^2^ = 0.2, *P*-value = 0.68, OR = 1.1, 95% CI = 0.77, 1.49) during the pandemic than their male counterparts. On the other hand, males were equally likely to use DHA to prevent ill health as females (χ^2^ = 0.02, P-value = 0.89, OR = 1.0, 95% CI = 0.72, 1.96). Further, pooled analysis showed significant variations in the proportion of study subjects who took DHS to prevent weight loss (χ^2^ = 14.4, P-value = 0.006), to prevent ill health (χ^2^ = 14.2, P-value = 0.01) and to hasten recovery from illness (χ^2^ = 24.9, P-value = 0.0001).

**Fig. 4:**
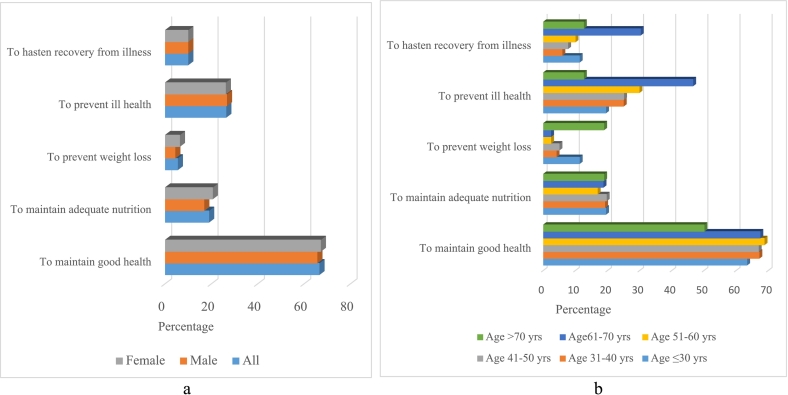
a, b. Reasons for the consumption of DHS during Covid-19 pandemic in Nigeria relative to gender (a) and age group (b) *Females were 1½ times, 1.3 times and about 1.1 times more likely to use DHS to prevent weight loss (χ*^*2*^*=1.2, P-value=0.27, OR=1.5, 95% CI=0.73, 3.09), to maintain adequate nutrition (χ*^*2*^*=1.4, P-value=0.24, OR=1.3, 95% CI=0.85, 1.91) and to maintain good health (χ*^*2*^*=0.17, P-value=0.68, OR=1.2, 95% CI=0.77, 1.49) during the pandemic than males. Males were marginally more likely to use DHA to prevent ill health than females (χ*^*2*^*=0.02, P-value=0.89, OR=1.0, 95% CI=0.72, 1.96). Pooled analysis showed significant variations proportion of study subjects who took DHS to prevent weight loss (χ*^*2*^*=14.4, P-value=0.006), to prevent ill health (χ*^*2*^*=14.2, P-value=0.01) and to hasten recovery from illness (χ*^*2*^*=24.9, P-value=0.0001).*

## 4 Discussion

This cross-sectional study intended to evaluate Nigerians' perception, knowledge, and consumption pattern of dietary and herbal supplements for the prevention and treatment of COVID-19. Both a web-based survey and a face-to-face interview were conducted from April 1 to October 31, 2021. Covid-19 disrupted many of the global functions and its rapid spread linked with the accompanying morbidity and mortality caused panic on individual, community, and governmental levels. It was expected that, due to the poor health systems in Africa, the continent would witness a large number of deaths from the pandemic. This expectation made many Africans, irrespective of status, age, or residence, take precautions, including use of dietary supplements and herbal products to prevent or treat the infection. This study may be one of the first, if not the first, to explore the prevalence and determinants of DHS use among indigenous Nigerians during the COVID-19 pandemic. Over half of the study participants (55.3%) were aged 31–50 years, unlike the study of Mohsen et al. [32] in which younger age group of 18–24 years formed almost half of the respondents. Majority of the respondents had attained secondary (26.4%) or tertiary (67.6%) level of education and only 6% had primary or no formal education a finding that is similar to what Alkhrashi reported in a similar study from Saudi Arabia [33] and what Radwan et al., [34] reported from UAR, but lower than what was reported in a Ghanaian study [29]. Further, a sizable proportion of Nigerians (65.9%) were aware that dietary supplements are necessary during infectious disease outbreak such as Covid-19, a finding that supports the view that dietary supplements can enhance people's health and strengthen their immunity [32]. There was no authentic data on prevalence of supplements consumption prior to Covid-19 pandemic, though it is however speculated that the prevalence was low, going by the example of the Ghanaian report [29]. This study nevertheless shows that the prevalence of supplement use during the pandemic was 88.5%, more among the females than among the males, more in older than younger age groups, more among those with higher educational status than among those with educational status lower than tertiary and more among married than among single or separated or widowed. This is in consonance with other studies which stated that consumption of dietary supplements is more among females, older people, and those with high education [[35], [36], [37]]. Further, this finding agrees with data from Saudi Arabia [38] Poland [31] and some other European countries [39] that there was a dramatic increase in the population who consumed dietary supplements or herbal products during Covid-19 pandemic. That there was a very high consumption of DHS during the COVID-19 pandemic may be as a result of contagiousness of the disease, the rapidity with which it was spreading across the globe, non-availability of any remedy that is accessible to the people, poor health system, growing burden of Covid-19 infection, distrust in the vaccine that was eventually imported, misinformation and disinformation and people's general disturbance, aggravation, frustration and urgency to be responsible for their health. The wide availability and easy accessibility of DHS in many Nigerian pharmaceutical stores, chemists, and open markets, without the approval of any supervisory authority, could be factors that brought about a rise in consumption of Supplements and herbal products. Another major finding was that majority (41.2%) of study subjects took dietary supplements daily. Surprisingly, daily consumption of dietary supplements was also more among females, those aged 41–50 years, the highly educated, the employed as well as married study participants. However, this finding was lower than the 66.5% study subjects reported in Riyadh to be consuming DHS daily [33] but higher than the 25.5% participants in a Sri Lankan study that indicated regular use of supplements [40]. A key finding is that Vitamin C was the most consumed dietary supplement by participants (53.6%) in this survey, especially among those aged 41–50 years, highly educated, employed and single individuals. This finding was higher than the 46% use of vitamin C reported from Sri Lanka [40] and the 42.1% use in Lebanon [32] but lower than the 84.5% use in UAR [34]. This is probably because research suggests that vitamin C probably improves immune response and reduce symptoms of some respiratory illnesses, like the common cold and flu [41,42]. Undeniably, several studies have documented the antioxidant and anti-viral effects of vitamin C [[42], [43], [44], [45], [46]]. It is pertinent to say, however, that proof of vitamin C efficacy on its efficacy in the therapeutic management of respiratory conditions is however ambiguous. Whereas some studies reported hardly any impact, others suggest that certain dosage of vitamin C is therapeutic, has a wide safety margin and is affordable for the management of common cold [47,48] though its impact on Covid-19 is still being investigated and remains to be proven [44,49,50]. Other reported vitamins consumed during Covid-19 pandemic in Nigeria were Multivitamin (33.6%), Folic acid (18.5%), vitamin B complex (16.6%), vitamin D (12.3%) and vitamin E (2.5%). The overall 12.3% proportion of those who consumed Vitamin D in this current study is higher than the 9.5% reported from Riyadh [33] or the 8.6% reported from Sri Lanka [40]. Vitamin D deficiency has been associated with COVID-19 high mortality rate in Asia, necessitating its supplementation to play a pivotal function in the prevention or treatment of the disease [51,52]. For this reason, vitamin D supplementation was given to elderly patients in France who were infected with COVID-19 and were less likely to exhibit the complications of the infection [53]. Vitamin D was also shown to be associated with reductions in Covid-19 infection in the population of US veterans [54]. Calcium was the most consumed mineral supplement in this current study. In all, 15.7% of the study population consumed Calcium, a figure that is higher than the 9.9% reported by Radwan in UAE [34]. Of the 645 participants in this study, only 4.2% consumed Selenium during the pandemic, a proportion that is less than the 7.0% reported from in Riyadh. The submission of Zhang and Liu [55] that dietary supplementation which include Selenium, Zinc, and Iron, among others, might enhance immunity against COVID-19 infection has been corroborated by the findings of Poos et al. [56]. In addition, Maares and Haase [57] reported the importance of Zinc for the preservation and development of adaptive and innate immune cells while Pae and Wu [58] reported that, in elderly people, low concentration of Zinc may be a risk factor for pneumonia since Zinc has anti-inflammatory and antioxidant properties and, Sharif et al. asserted that uptake of vitamin C, vitamin D and zinc are were significantly associated with the reduced risk of infection and severity of COVID-19 [59]. Another key finding in this study is that ginger was most consumed (25.9%) special plant-based supplements, ahead of garlic (18.3%), turmeric (15.8%), Moringa (9.2%), ginseng (3.6%) and herbal teas (8.2%) in the evaluated DHS practices during Covid-19 pandemic in Nigeria. This proportion of study subjects that consumed ginger during the pandemic in Nigeria was much less than the 56.4% who consumed it in United Arab Emirate [34] but higher than the 20% who took it in Lebanon [32], the 18.5% in Riyahd [33]. Studies have reported evidence of therapeutic effects of ginger in inflammatory condition in the lungs such as pneumonia, fibrosis and sepsis, conditions that are associated with high morbidity and mortality in Covid-19 infected people, though the mechanism of action of ginger in these situations is yet uncertain [[59], [60], [61], [62]]. Thota [63] also suggested that other plant-based, spicy, natural products such as turmeric and garlic evaluated in this study also have therapeutic values while Singh et al. [64] believed these products have natural immunity-booster properties because they have anti-viral, antinociceptive, anti-inflammatory, antipyretic, and anti-fatigue properties which can be harnessed in the effective management of COVID-19. The 18.3% proportion of this study's participants who consumed garlic (*Allium sativum*) during the pandemic was higher than the 4% who did so in Lebanon [32] but less than the 53.8% use reported from Riyadh [34]. Studies have put forward evidence that garlic has nutritional, and therapeutic properties [[64], [65], [66]]. Garlic is believed to possess anti-neoplastic, antibiotic, anti-hypertensive, antioxidant, and anti-lipemic actions probable because of it allicin and alliin which are sulphur-producing agents [67,68]. It was noted that the use of garlic as a treatment was also prominent in a previous epidemic [64]. However, these claims of the effectiveness of plant-based supplements and products for the management of Covid-19 still require further investigations due to lack of credible data.

This study also evaluated the sociodemographic determinants of DHS use for COVID-19 prevention or treatment in Nigeria. The results indicated that there was higher probability for females, older adults (especially those aged 41–50 and 61–70 years), widows and those with secondary education to like state that they consumed DHS to prevent or treat COVID-19. This finding aligns with what other studies have reported relative to age distribution [[69], [70], [71]] and gender disparity [[72], [73], [74], [75]] in the use of supplements, though some other studies presented a divergent view [34,38]. Majority (42%) of the study respondents depended on health workers as the main source of information on consumption of DHS during the pandemic in the country and only 13% relied on social media for such information. This may be due to uncertainty, conspirator theories and fake news that were being disseminated through the social media and people felt that their health workers could be trusted as being better informed. For example, Kouzy et al. [76] analyzed Twitter contents during the pandemic and discovered that approximately 25% of information concerning Covid-19 were misleading the public and Lelisho et al. [77] reported that use of social media has a substantial influence on the development of panic among people regarding the COVID-19 epidemic.

### 4.1 Study limitations

There are certain limitations in this study some of which need explanation. The first is that there were two sources of data (i) Google Form sent through the internet to all the six geo-political zones of Nigeria, and from which not enough response was received. This warranted the use of face-to-face interviews, with full precautions taken, in the Southwest geopolitical zone, mainly in Lagos and among people from various ethnic groups in the country. Therefore, the response in this study may not be nationally representative, though data from Lagos were from almost all ethnic groups in Nigeria. Another limitation is that convenient sampling was used which may have introduced bias into the study. Part of the data was collected digitally, through the internet as self-completed format. Thus, subjects may have misunderstood, misinterpreted, misread the questions, and had nobody from who to seek clarifications. In this context, those who were involved in DHS and interested in its use would have participated in the on-line Google Form questionnaire survey while other vulnerable population might have been excluded. The other part of the study was interviewer-mediated which may have introduced potential social desirability and interviewer biases. Moreover, it is important to note that the study population consisted of a relatively high proportion of women and those with higher education, but this is, to an extent, slightly comparable to the general population in Nigeria and which however may limit the generalizability of the study's findings. The relatively small sample size and cross-sectional survey design delimits causal associations between study variables. There are hardly any studies conducted in Nigeria or in Africa on the advantages and benefits of using dietary and herbal supplements for preventing and treating COVID-19 infection, which incapacitated this current study to compare its findings with those of other African countries. Wider, large-scale prospective studies, covering all the geo-political zones of the country and the Federal Capital Territory are required to determine the precise predictive value of findings in this current study.

## 5 Conclusion

This study evaluated the proportion of Nigerians who consumed dietary and herbal supplements such as vitamins (B complex, C, D, E Folic acid and Multivitamin), trace elements (Selenium, Calcium, Zinc and Iron), herbal teas, and some natural herbal products (Garlic, Ginger, Turmeric, Moringa and Ginseng) to boost their immunity during the COVID-19 pandemic in the country. Approximately 9 in 10 of the study population took dietary and herbal supplements strengthen their immune status. Although age grouping was marginally associated with supplement intake, it was significantly linked with frequency of consumption of DHS during the pandemic as was occupational status. In general, increased intake of DHS was more common in older age group widows, those with secondary education and those not employed. Vitamin C was the micronutrient consumed by a significant proportion of the study participants, mostly among those aged 51–60 years. Common reasons why study respondents consumed DHS during the Covid-19 pandemic in the country were maintain good health and prevent ill health. Few said it was to hasten recovery from illness (9.8%).

## Contributors

SH, BMA, ND, OK, AS, and OA conceived and designed the initial research questions and coordinated the data collection. BMA, SH, and OK had full access to the study's data and take responsibility for the integrity and accuracy of the data analysis. BM and SH completed the statistical analysis and supervised the conduct of the research. BMA and SH drafted the final paper. BMA, SH, OK, ND, AS and OA critically reviewed the write up of the manuscript. All authors approved the final version of the manuscript and agreed to be accountable for all aspects of the work.

## Funding

This research was funded by the Nigerian Institute of Medical Research Grant No: **NMG-CIF-38-0056.s**.

## Declaration of Competing Interest

The authors declare that they have no known competing financial interests or personal relationships that could have appeared to influence the work reported in this paper.
